# Internal and External Reinforcement of Concrete Members by Use of Shape Memory Alloy and Fiber Reinforced Polymers under Cyclic Loading—A Review

**DOI:** 10.3390/polym10040376

**Published:** 2018-03-28

**Authors:** Azadeh Parvin, Janet Raad

**Affiliations:** Department of Civil and Environmental Engineering, The University of Toledo, Toledo, OH 43606, USA; janet.raad@rockets.utoledo.edu

**Keywords:** near-surface-mounted (NSM), joints, fiber reinforced polymer (FRP), FRP bars, external FRP strengthening, shape memory alloy (SMA), concrete

## Abstract

This paper presents a review of recent studies on reinforced concrete (RC) structural components, such as beam-column joints (BCJs). These members are internally or externally reinforced with corrosion free shape memory alloy (SMA), fiber reinforced polymers (FRP), or a combination of the two materials. Bonded FRP sheets or near surface mounted (NSM) FRP bars are used in external strengthening cases. The use of FRP and SMA materials in RC structures can offer great potential benefits including lifetime cost saving, durability, safety, and post-earthquake serviceability for RC structures. Although FRP materials are well known for their corrosion resistance, high strength-to-weight ratios, ease of application, and constructability; SMA materials as reinforcement allow the structures to regain their original shape after the termination of the load without any permanent large residual deformation. In summary, the presented literature review provides an insight into the ongoing research on the use of these materials for retrofitting or strengthening of RC structural components and the trends for future research in this area. The cost and durability are also discussed.

## 1. Introduction

One of the causes of deterioration of RC structures is due to the corrosion of embedded reinforcing bars followed by the formation of cracks in concrete resulting in putting the structures out of service. For many decades, the problem of corrosion has remained an alarming issue. Likewise, the damage caused due to earthquakes is another severe issue posing a threat to the safety of structures, particularly for those built prior to 1970s, which were designed for the gravity loads with no seismic provisions. Since then, changes were made to the design code to prevent the newly built structures from collapse under seismic event [1]. However, it is essential to retrofit the non-seismically designed deficient structures to prevent future damages by severe earthquakes. For many years the retrofitting of buildings was carried out by traditional methods such as concrete and steel jacketing to provide additional needed strength, until the use of innovative materials. More recently, many studies around the world have been directed towards the use of FRP, due to their light weight, corrosion resistance, and ease of installation as compared to the conventional methods. Numerous researchers have worked on the use of FRP sheets as external reinforcement for confinement, shear, and flexural strengthening [1,2,3,4,5,6]. However, only few researchers have started to work on the concept of using FRP as internal reinforcement for BCJs, which are critical components, and their failure could result in the total collapse of the structures [7,8,9,10,11,12].

On the other hand, super-elastic (SE) shape memory alloys (SMAs) are the type of materials, capable of undergoing large deformations and returning to their original shape and position upon the removal of applied forces. As a result, the use of SMAs as structural reinforcement reduces the damage to the overall structures even after being hit by an earthquake. SMAs are used in the form of sheets and strips. However, few researchers have recently used SMA as internal reinforcement bars in the plastic hinge regions where the damage can cause failure of the whole structure [13,14,15]. Such advantages from both FRP and SMA, have paved the way to start using them together either as a hybrid or composite materials for strengthening RC members both externally and internally. Some studies performed on the BCJs consider these hybrid or composite materials to improve the overall performance of structures [16,17,18].

FRP and SMA materials offer a viable reinforcement solution that is noncorrosive as oppose to traditional steel reinforcement which decays over time when exposed to harsh and alkaline environment. The aim of this paper is to provide an insight into the performance of corrosion free concrete BCJs reinforced with FRP, SMA, and a new composite material made out of FRP and SMA. It is called hybrid when the joint has SMA bars in a region and FRP bars in another one, while it is called composite when the bars are made of a mixture of both FRP and SMA materials.

## 2. Non-Corrosive Reinforcement Materials for Concrete Structures 

In recent years the use of fiber reinforced polymers (FRP) and shape memory alloys (SMA) in RC structures has gained popularity due to their superior material properties and resistance to corrosion. The application of these smart materials could easily replace the traditional steel reinforcement in newly built concrete structures, or can be used for the retrofit of existing structures. BCJs are one of the most critical assemblies in the structural system. Furthermore, investigations on BCJs are more spar as compared to beams or columns. In the following sections BCJs reinforced with SMA, FRP and hybrid or composite SMA-FRP as internal and FRP as external reinforcement are discussed.

### 2.1. Beam-Column Joints Internally Reinforced with FRP Bars

Investigations on BCJs internally reinforced with FRP bars are limited as oppose to more common method of external FRP reinforcement. Most of these studies have commonly used glass fiber reinforced polymer (GFRP) material for reinforcement bars (Table 1). Said and Nehdi [7] tested two full-scale BCJs, one reinforced with GFRP grid and one was reinforced with steel bars and stirrups under cyclic loading. Although the 3% minimum drift ratio requirement of ductile frame was achieved in the GFRP reinforced joint, lower stiffness and energy dissipation were observed due to elastic behavior of the GFRP material as compared to the steel reinforced joint. Furthermore, the beam tip load was as high as that of the steel reinforced joint. Because of the low stiffness of GFRP bars, the joint failure was due to the brittle behavior that led to the rupture of two bottom GFRP longitudinal bars.

On the other hand, Saravanan and Kumaran [8] conducted experiments on eighteen BCJs reinforced with GFRP stirrups and bars, followed by finite element analysis study. Variables considered were bar types (threaded, sand-coated, and grooved), beam and column reinforcement ratios, concrete strength, and joint aspect ratio. Furthermore, the influence of GFRP stirrups on the joint shear strength was investigated and a design equation to predict the joint shear strength was proposed. Their study showed that compared to the BCJs reinforced with traditional steel bars, the GFRP sand-coated reinforcement improved the load carrying capacity by almost 5%, but more importantly the deformation capacity increased by 30% to 50%. Moreover, the presence of stirrups in the joint area was able to move the failure from the joint core to the beam-column interface. 

Two other experiments in 2011 were conducted by Mady et al. [9] and Hassaballa et al. [10] on full-scale concrete BCJs reinforced with GFRP bars and stirrups when subjected to seismic loading to explore the influence of GFRP reinforcement on the behavior of the joints. Mady et al. [9] had the longitudinal and transverse reinforcement material types (steel and GFRP), and beam longitudinal reinforcement ratios as parameters. While, Hassaballa et al.’s [10] variables of study were longitudinal and transverse reinforcement materials (steel and GFRP) and the beam’s longitudinal bar details (with hooks, straight, or straight with extension into a beam stub). Both experiments had one reference specimen (longitudinal and transversal steel reinforcement), one specimen reinforced with GFRP bars and steel stirrups, and the rest were reinforced with GFRP bars and stirrups. Mady et al. [9] revealed that the increase in the GFRP longitudinal bars reinforcement ratio would result in higher energy dissipation in the joint. Their findings of using steel instead of GFRP stirrups would result in an increase of dissipated energy. In Hassaballa et al.’s study [10], even though both GFRP reinforced and control joints failed in shear, the failure mode of GFRP reinforced joint with extended stub was observed by the formation of plastic hinge away from the column face which satisfies the design capacity concept (weak beam-strong column). Furthermore, the BCJs with GFRP bars as internal reinforcements of both experiments [9,10], sustained 4% storey drift ratio safely with no considerable damage or residual strains. Thus, the BCJ could retain its original shape upon removal of the seismic loads up to this drift ratio. Moreover, all joints exceeded their individual design capacity by an average of 9%. Based on Mady et al. [9] and Hassaballa et al.’s [10] investigations, one can conclude that the BCJs reinforced with longitudinal and transverse GFRP reinforcement, usually provide lower energy dissipation as compared to steel reinforced BCJ. Table 1 shows additional details of the experiments and results.

Similarly, Hasaballa and El-Salakawy [11] tested six full-scale GFRP-reinforced exterior BCJ prototypes under seismic loading. The parameters of their study were concrete strength, and shear stress level in the joint. Diagonal shear cracks were observed in some of the joint specimens. For the same shear stress level, similar strains were recorded in the joints stirrups at failure even though the failure occurred at different drift ratios. Furthermore, the energy dissipation and ductility were higher for the joints that had lower concrete strength as compared to those with higher concrete strength. Therefore, the joints with higher energy dissipation were able to regain their original shape after unloading. Further details and results are summarized in Table 1.

Similar to the latter reference, Ghomi and El-Salakawy [12] performed experiments on six full-scale BCJs reinforced internally with GFRP bars and stirrups under seismic loading. The joints had lateral beams on all four sides of the column. The variables considered were reinforcement materials (steel and GFRP), presence of lateral beams, joint shear stress level, and end anchorage of beam longitudinal bars (headed-end, and bent bars). Their findings revealed that in some cases, GFRP-RC BCJs confined with lateral beams provided nonlinear behavior and non-brittle failure, despite the expected linear behavior from FRP-RC structures. This was also due to high shear stress level in some joints, where at the same drift ratios they could dissipate more energy as compared to those joints with lower shear stress level. Furthermore, both methods of anchorages performed well with shear stress level of 1.1 √*f*_c_^′^ or higher.

### 2.2. Beam-Column Joints Internally Reinforced with SMA-Steel Hybrid Bars

Despite the advantages of SMA materials such as, energy dissipation, and self-centering, limited studies have been performed on the use of SMA bars in BCJs. Furthermore, due to their cost, SMA bars have particularly been used in crucial regions of BCJs at the location of plastic hinges, while the other regions are typically reinforced with conventional steel bars. This type of bar reinforcement arrangement is referred to as hybrid SMA-steel. Researchers have noted that reinforcing structures with SMA bars provided remarkable results in terms of capability of regaining their original shape without any residual displacement after the load is diminished (e.g., [13,14,15]). Alam et al. [13] developed a finite element model based on an analytical model that has been done previously. The accuracy of the finite element model was verified by comparing their results with two experiments [14,15]. One involved a bridge pier reinforced with SMA bars and spirals [14], and the other one was BCJ reinforced with SMA bars in the plastic hinge region coupled by steel couplers with steel bars in other regions [15]. Both specimens were subjected to cyclic loading. The results of both experimental studies on the SMA reinforced BCJ and the bridge pier showed that they were able to recover most of their post-yield deformation; thus, the repair requirements would be minimal [14,15]. Consequently, Alam et al. were able to identify the discrepancies in moment-curvature relationships of the BCJs to predict the location of the plastic hinge, crack width, and bond-slip relationship for SMA reinforced joints when subjected to seismic loading. The numerical results showed that finite element analysis can predict the moment-rotation and load-displacement curves with reasonable accuracy [13].

### 2.3. Beam-Column Joints Internally Reinforced with Hybrid or Composite SMA-FRP Bars

SMA-FRP hybrid or composite reinforcement of concrete structures offers an advantage to efficiently strengthen the most critical locations in structures like BCJs. Experimental studies conducted by Zafar and Andrawes [16,17] were mainly on the application of SMA-FRP composite as an innovative material to reinforce RC moment resisting frames (MRFs) in order to enhance their behavior under seismic conditions and to reduce their residual drifts after the occurrence of an earthquake. They observed that due to higher initial stiffness, the frame reinforced with steel experienced lower inter-storey drifts (IDs) when compared to those reinforced with SMA-GFRP and GFRP. The frame with steel reinforcement undergone 84% and 62% more residual IDs when compared to those reinforced with SMA-GFRP and GFRP bars, respectively. For the same peak ground acceleration (PGA) value, the frame reinforced with SMA-FRP was capable of dissipating more energy as compared to the GFRP frame, as well as it could resist 51% higher seismic demand (PGA) when subjected to different seismic events as compared to the frame reinforced with steel bars [17]. SMA-FRP reinforced frames also exhibited almost negligible residual ID values as compared to the GFRP and steel reinforced frames. The study further revealed that the application of composite SMA-FRP bars in the zones of plastic hinge of MRFs did not only improve ductility considerably, but also residual drifts and energy dissipation were improved when compared with the frames reinforced with GFRP only at the same drift ratio. Consequently, the overall performance of the frames under seismic loading conditions was enhanced.

Another study was conducted using SE-SMA bars in the zone of plastic hinge and FRP in other zones of a steel-free BCJ under cyclic loading [18]. It was found that the coupled SE SMA-FRP bars formed a force versus displacement hysteresis curve for the BCJ, similar to that of the steel RC joint with lower stiffness and comparable residual drift. The utilization of SMA at plastic hinge region of the BCJ was supposed to considerably reduce the residual drift due to its quality of being highly super-elastic. However, the observed distortion was likely because of the slippage of the FRP bar inside the couplers. Nevertheless, the BCJ reinforced with SMA in the core region had the capability to withstand 89% of its load carrying capacity beyond the ultimate limit. Furthermore, in the case of steel-RC BCJ specimen the plastic hinge formed at the column face. Conversely, by using SE-SMA in the joint region, the plastic hinge zone was successfully transferred away from the face of column by a distance equal to approximately one fourth of the beam depth.

### 2.4. Beam-Column Joints Externally Reinforced with NSM FRP or SMA Bars

The use of near surface mounted (NSM) FRP or SMA reinforcement has become an attractive method for enhancing the flexural and shear capacities of RC beams or columns. The process of NSM FRP or SMA technique starts with cutting grooves on the surface of concrete elements, without any need for surface preparation. Then, the SMA or FRP bars are installed in the grooves using epoxy filling. This method is less time consuming as compared to other external reinforcement techniques.

Several studies have been performed on external reinforcement of beams and columns with the NSM technique using FRP or SMA bars. However, to the best of authors’ knowledge, NSM technology with bars has not been fully extended to BCJs. One study by Prota et al. [19] examined external RC BCJs under seismic loading. Their strengthening technique involved the extension of beam NSM FRP bars to the back of the joint region followed by externally bonded FRP laminates as anchorage. The test results showed a change in the mode of failure from shear in the joint core to flexure in the beam.

Recently, a modified NSM CFRP-strengthening technique of RC BCJs has been investigated experimentally [20]. This technique is based on making grooves in the joint region, filling the grooves with epoxy, and externally bonding the region with CFRP sheets instead of embedding the CFRP in the grooves. The effectiveness of this modified technique along with FRP fans at the termination point of FRP sheets was examined. It was found that the external bonding FRP reinforcement on grooves (EBROG) significantly enhanced their strength, stiffness, ductility (enhancement of 54%, 84%, and 74% as compared to the control counterpart specimen, respectively), pinching width ratio, and energy dissipation (retrofitted with CFRP sheets and fans together increased twice of that of the control one). The combination of the external bonding of FRP on grooves and FRP fans stopped the debonding and delayed the brittle failure. Additionally, the plastic hinge was moved away from the joint to the beam.

### 2.5. Beam-Column Joints Externally Reinforced with FRP Sheets or Straps

In the recent years, the FRP materials have been widely used to externally reinforce and retrofit the RC structures. The aim of using these materials is either to increase the structural load capacity or to repair the deteriorated structures along with the application of other materials such as mortars. The increase in the load capacity maybe necessary in order to address new code requirements, errors in the design or construction process, or to sustain an extra live load. In external reinforcement, the propagation of microcracks is a factor that plays an important role in affecting the bond strength. To that end, multiscale analytical models have been developed to predict either the delayed debonding of FRP external strengthening or the bond lifetime e.g., [21,22]. However, there are new methods to improve the bond of external reinforcement such as vacuum applications, stud shear connectors, and anchor spikes [23,24]. In this section, we shed the light particularly on some studies that have used FRP sheets or straps to strengthen the most critical structural element namely, the BCJ [1,5,6,25,26,27,28,29,30,31,32,33,34,35,36,37,38,39,40].

Few experimental investigations on BCJs have used steel anchorages for the FRP sheets [25,26,37,40]. Two of these studies were performed on the same size BCJs under the same loading conditions using different number of FRP layers and different steel anchorage configurations [25,26]. Ghobarah and Said [25] did an experiment on four non-seismically designed RC BCJs (with no shear reinforcement in the joint core). Two of the specimens were considered as control joints and were subjected to quasi static cyclic loading. After this phase of the experiment, the two specimens were repaired using unidirectional and bidirectional U-shaped and X-shaped GFRP sheets on the columns at the joint core location only, and the other two undamaged joints were strengthened with the same retrofit configurations as the damaged ones. Then, the four specimens were subjected again to quasi static cyclic loading to find the effectiveness of rehabilitation. Steel plates and threaded steel rods were used as anchorages for the GFRP sheets. More details of GFRP sheets and material properties are provided in Table 2. The comparison between the control and strengthened specimens showed that, the GFRP helped improve the shear capacity of the joints. Additionally, the ductility and the energy dissipation were increased by approximately 62% and 72%, respectively. The GFRP strengthening was successful in delaying the shear failure, and in some cases, the failure mode was transferred from shear failure to flexural hinge in the beam. Furthermore, El-Amoury and Ghobarah [26] conducted another experiment on the same size BCJs under the same loading conditions but with different number of GFRP layers and steel anchorage configurations. Besides GFRP column confinement, L-shaped GFRP sheets were added at the bottom face of the beam and column joints followed-up by two different anchorage systems. The first system was somewhat similar to that of Ghobarah and Said [25] study using steel plates with threaded steel rods on the column at the joint core, except a steel angle was added at the bottom of BCJ core. In the second system, two U-shaped steel plates were applied to anchor the extended GFRP sheets on beams in order to avoid the bond slip of steel bars and the debonding of GFRP sheets. Following up with the latter experiments [25,26], Ghobarah and Al-Emoury [37] had two groups of BCJ specimens. In the first group, same anchorage systems [25,26] were employed but the GFRP sheets were replaced with CFRP. In the second group, different configurations of anchorage systems were incorporated involving the use of additional steel rods and plates. In [25,26,37], the brittle shear failure of the joints was eliminated, and load-carrying capacity, ductility and energy dissipation were improved. Specifically, the new anchorage system and GFRP configuration [26] delayed the debonding of GFRP sheets, as well as the slippage of beam’s top reinforcement. Moreover, the use of rods [37] led to an improvement in the anchorage system conditions where the tensile strength could be fully achieved (Table 2).

Similarly, a more recent study was conducted on seismically deficient RC BCJs using steel plates with different shapes (U-shaped, L-shaped steel angles, and horizontal plates) in addition to threaded steel rods, to anchor the CFRP sheets (uniaxial, quadriaxial) in strengthening either damaged or undamaged joints [40]. The joints were tested under cyclic loading to investigate the effectiveness of CFRP configurations (X-shaped, U-shaped, and horizontal), the anchorages methods using steel elements, and different internal steel reinforcement ratios. The results obtained from the experiment indicated that FRP-retrofitted members regained their strength and achieved a higher ductility than its control counterparts. It was also noted that the use of L-shaped steel angles on all corners of the joint provided confinement resulting in high level of displacement capacity up to 75% (Table 2).

The same idea was applied by Al-Salloum and Almusallam [28] in their experiment on RC BCJs designed under gravity load with pre-1970s deficient reinforcement details. Four half-scale joints, two controls and two strengthened with CFRP, were tested to study the efficiency of the CFRP sheets in upgrading the shear strength and ductility when subjected to seismic loads. Two different schemes were employed to strengthen the joints. In the first scheme, CFRP sheets were epoxy bonded to the BCJ regions. In the second scheme, sheets were epoxy bonded to the joint core only and mechanical anchorages were also provided to prevent any debonding. Furthermore, the damaged control specimens were repaired by filling the cracks with epoxy and were wrapped with CFRP sheets and tested again. Hence, a total of six specimens (two controls; two strengthened; and two repaired) were considered. It was observed that although in the first scheme, the CFRP reinforcement was extended from the joint to the beam and the column, the debonding happened due to the lack of anchorages. However, the provided anchorages in the second scheme were able to move the failure from the joint area to the beam. Furthermore, both schemes were successful in enhancing the strength and ductility as well as providing stiffness against shear distortion for the joints. Further results are provided in Table 2.

Most experimental studies have been performed on scaled-down with few on full-scaled BCJs (e.g., [1,6,29,32,38,39]). Six full-scale non-seismically designed beam-column joints were strengthened with CFRP sheets and subjected to cyclic loading [1]. The parameters of this study were the number of CFRP layers, CFRP sheets configurations, and the effects of lap splice and axial load on the column. Various CFRP configurations were applied to the joints. Based on the experimental results, it was noted that the increase in column’s axial load improved the stiffness and lateral load capacity in both control and CFRP-strengthened joints. However, the CFRP rehabilitation enhanced the overall structural performance of the joints. Increase in number of CFRP layers resulted in higher stiffness, lateral load, and energy dissipation capacities (Table 3). Various anchorage systems stopped the debonding of CFRP sheets and the slippage of shortly embedded bottom bars in the beam was avoided.

Another study on full-scale joints evaluated the capacity of RC damaged joints representing post-earthquake situation [6]. The deficient joints were repaired with high strength concrete and then wrapped with CFRP sheets. It was found that the replacement of the concrete in the joint core with high strength concrete enhanced the shear strength of the joints up to 44% as compared to the control specimen. In addition, the CFRP strengthening was adequate to develop the full plastic capacity of the joint and increased the strength up to 69% (Table 3).

Additionally, two full-scale RC BCJs subjected to cyclic loading on the beam tip were tested by Vatani-Oskouei [29]. The two specimens were then repaired and strengthened with CFRP sheets along with steel angles added to the top and bottom faces of the beam. The results indicated that adding the CFRP sheets to the damaged joints helped to increase the load-carrying capacity, the amount of energy dissipated by the joints and the ductility. Furthermore, the failure location moved from the beam-column interface to the beam. However, the failure was due to the rupture and debonding of CFRP sheets. It is worth noting that injecting epoxy into the cracks did not enhance the overall behavior of the specimens. Details and results are noted in Table 3.

Another full-scale test was performed on a non-seismic BCJ subjected to reverse cyclic loading which was repaired and strengthened by CFRP sheets around the joint core and tested again [32]. The specimen was built using low compressive strength of concrete (8.5 MPa) and plain reinforcement bars. Upon strengthening, the failure mode was changed from the joint shear failure to the rupture of CFRP sheets. It was noted that increasing the number of CFRP diagonal sheets around the joint, might delay the CFRP rupture which in turns it would result in higher drift ratios and horizontal load capacities (see Table 3 for more details).

In another study [38], six full-scale corner BCJs including slab were tested under cyclic loading to study the influence of several parameters on their behavior. Variables considered were the presence of CFRP joint strengthening below the slab level, fibers orientation, CFRP thickness, column confinement, and concrete compressive strength. The results obtained indicated that, the use of a suitable amount of CFRP reinforcement helped to increase the strength of the joint while avoiding the full debonding of CFRP ends. Higher concrete strength resulted in higher peak joint strength. Moreover, various combined modes of failure were observed depending on the strengthening configurations, i.e., CFRP debonding accompanied with joint shear failure, CFRP debonding accompanied with column flexural hinging, and CFRP rupture with column flexural hinging (Table 3).

A new idea was employed by Di Ludovico et al. to test a full-scale RC structure in order to understand the global behavior of RC frame when BCJs were retrofitted by GFRP laminates [39]. The variables in this study were, the level of PGA (0.2, 0.3), the GFRP configuration (uniaxial, quadriaxial), and the column cross-section dimensions and confinement. It was noted that, although the confinement of the columns by GFRP laminates did not significantly increase the strength; however, the rotational plastic hinge capacity extremely improved. It was concluded that the use of GFRP for retrofitting RC joints would considerably improve the seismic performance of the frame structure. Further details are provided in Table 3. 

In addition to large-scale testing, due to budgetary constraints, and laboratory space and equipment limitations, many researchers performed experiments on scaled-down BCJs, (e.g., [31,33,36]. In one experiment test was carried on one seismic (S) and four non-seismic (NS) 2/3-scale RC BCJs [31]. The NS joints were subjected to reversed cyclic loading then they were repaired and strengthened with various CFRP laminates shapes (U-shaped, L-shaped, sheets, and wraps) and tested again. The results of the four NS strengthened damaged joints were compared to the seismically designed joint. The repaired and strengthened specimens could achieve load-carrying capacity equal to or higher than that of the seismic one. However, the initial stiffness of the strengthened specimens was lower than the seismically designed joint due to the existing cracks. Based on the damage index assessment achievement, it was noted that the use of CFRP laminates is effective up to the repair-ability performance level in order to improve the joints seismic behavior. More Details about the specimens and results are provided in Table 4. In another experiment, Le-Trung et al. [33] tested RC BCJs strengthened with FRP sheets under cyclic loading. The experiment, performed on 1/3-scale, exterior joints consisted of one non-seismically (NS) and one seismically (SD) designed specimens and six non-seismically designed specimens (RNS) retrofitted with CFRP sheets. The parameters of the study were CFRP sheet thickness and configurations (T, L, X-shaped and strip combinations). It was observed that the NS BCJ failed in brittle manner in shear with significant damage, while the SD joint showed more of a flexural failure in the beam indicating a ductile behavior. Furthermore, the use of CFRP sheets for the non-seismic joints resulted in the lateral strength and ductility improvement. The X-shaped, and the combination of T-shaped/L-shaped/column strips configurations outperformed other strengthened joints in terms of strength and ductility. The failure mode changed from shear in the joint core to flexural failure in the beam (Table 4).

Another experimental study was conducted on eighteen 2/3-scale RC BCJs to examine the effectiveness of large number of parameters including presence of mechanical anchorages, area fraction of FRP, distribution of FRP between the beam and the column, column axial load, internal joint steel reinforcement, initial damage, carbon versus glass fibers, sheets versus strips, number of FRP sheets or strips, and the effect of transverse beams [36]. The results obtained from the experiment indicated an increase in the effectiveness of both strips and sheets when using mechanical anchorages, an increase of 30% was achieved in terms of strength, while the energy increased by 40%. The most effective axial load on the column was 2.5 higher than the initial loading. The strength and energy dissipation increased with higher number of FRP layers. Additionally, it was observed that increasing the area fraction of FRP in both the columns and beams led to an increase in the strength and the energy dissipation in a comparable amount to those where only the area fraction of the beam was increased. It was also noticed that, the use of GFRP was slightly better than CFRP in increasing the strength, while it achieved higher increase in terms of energy dissipation when compared to CFRP. The presence of transverse beam diminished the dependency on FRP sheets in terms of strength and energy dissipation, more results are illustrated in Table 4.

In the experimental studies discussed so far, only Antonopoulos and Triantafillou [36] had the FRP material type as a variable. However, other experiments were conducted on exterior BCJs strengthened with CFRP or GFRP materials [27], and interior BCJs strengthened with the combination of CFRP and GFRP sheets [5]. Mukherjee and Joshi [27] investigated two different types of joints, non-seismic (non-ductile), and seismic (ductile). Two FRP-strengthening schemes were proposed. Both schemes have seismic and non-seismic joints. For the first scheme, the CFRP or GFRP sheets were used on the transverse beams and the column around the joint core to strengthen the joints. In the second scheme, CFRP plates were installed on the transverse beams and wrapped with CFRP sheets to improve the bending stiffness. Additional joint details and material properties are shown in Table 5. The joints were subjected to cyclic loading and then damaged specimens were repaired by replacing the loose concrete with epoxy and strengthened with CFRP or GFRP sheets. The results showed that GFRP and CFRP strengthening enhanced the lateral load capacity, ductility, and energy dissipation. The increase in ultimate deflection was an average of 33% as compared to the control counterpart specimen; an indicative of higher ductility. Also, the increase in energy dissipation in the non-seismic strengthened joints, compared to control counterpart, was 216%, 104.3%, 111.5%, and 60.6% for the BCJs reinforced with double GFRP sheets, single CFRP sheets, double CFRP sheets, and CFRP plates, respectively. For the seismic joints, the increase in energy dissipation was 76.4%, 97.5%, 151.48%, and 119.8% for the BCJs reinforced with double GFRP sheets, single GFRP sheets, double CFRP sheets, and CFRP plates, respectively compared to control counterpart. Furthermore, using two layers of CFRP or GFRP led to higher load capacity for both seismic and non-seismic joints. The pullout of the CFRP and steel reinforcement was the mode of failure for the joints strengthened with CFRP plates; however, the yield load was enhanced considerably. Using the CFRP as oppose to GFRP sheets helped the joints to exhibit higher stiffness (Table 5). For the interior joints experiment, four RC non-seismically interior beam-wide column joints subjected to cyclic loading were strengthened with two schemes [5]. The joints were repaired by injecting epoxy in the cracks and then strengthened with GFRP sheets (scheme 1) and CFRP and GFRP sheets (scheme 2) and tested again. Fiber anchors were also used to prevent the debonding. The four specimens were divided into two series based on the dimensions of the joints (details in Table 5). The results of the damaged specimens were compared with those of control counterparts. It was observed that increasing the axial load applied on the column helped to close the cracks which occurred on the side face of the column in some of the specimens. Although the anchors were provided to prevent delamination of FRP sheets, the debonding occurred in all specimens. Moreover, scheme 1 could recover the original stiffness and the strength capacity and the energy dissipation was increased by an average of 74% as compared to the control counterpart. Similar improvement was observed in scheme 2. The energy dissipation capacity was enhanced by 61% to 92% depending on the specimen size. The latter result indicated that the use of CFRP sheets was better in confining the joint than GFRP although the failure mode for both schemes was debonding of FRP sheets (Table 5).

On the other hand, Analytical models have been developed by researchers on CFRP strengthening BCJs based on experimental results of their own or others [30,34,35]. 

Based on experimental studies [29,30,31,32,33,34,35,36], Hadi and Tran [30] performed experimental and analytical studies on four RC non-seismic BCJs subjected to reversed cyclic loading. One joint was kept as a control specimen, while the other three were strengthened externally with CFRP wraps. The shape of the columns was modified from square to circular sections by using concrete cover followed by CFRP wrapping. Experimental results showed that the columns shape modification helped the confinement effectiveness of the CFRP wraps (Table 3). As a result, the shear capacity and the overall performance of the joints were improved and debonding and/or bulging of FRP wrap from the concrete surface were eliminated. Due to the increase in CFRP ratios, the failure changed from joint core shear to flexure in the beam, and the plastic hinge moved further away from the joint to the beam (Table 3). To predict the shear capacity an analytical model was developed and validated with the present CFRP-strengthened joints results [33] and 32 other joints in the existing literature. The analytical model proved to be suitable for practical applications. Additionally, the shear capacity of RC members has also been predicted in the case of RC member subjected to concentrated load [41].

Another analytical model was developed by Le-Trung et al. [34] for the joints with the same CFRP configurations of Le-Trung et al. [33], using the DRAIN-2DX program. The analytical model could accurately predict the CFRP strengthened joint behavior by taking into account joint shear behavior, bond slip of longitudinal beam reinforcement, and the effects of anchorage at the ends of the attached CFRP sheets.

Although few studies have examined analytical models of FRP-strengthened RC BCJs [30,33,34], there is a lack of simple and generalized formulations to predict the shear capacity. Del-Vecchio et al. [35] collected experimental data from large-scale experiments performed on RC joints [1,27,28,30,36,37,38,39,40]. Their study examines a new and simplified model to predict the shear capacity and study the effectiveness of the FRP in strengthening deficient corner BCJs subjected to severe seismic loading. The parameters included in the model were defined based on the effect of externally bonded FRP systems used in large number of data set obtained from existing experimental work in the literature. The proposed formulation had good agreement with the experimental results in terms of average effective strain, and could predict the shear strength of corner beam-column joint strengthened with FRP while taking into consideration the effect of all parameters of experimental studies used in their analytical model. Furthermore, the model was able to avoid the brittle shear failure of the joint by knowing the amount of FRP reinforcement needed.

## 3. Durability and Cost Associated with FRP SMA Reinforcement

One of the criteria to assess the durability of RC members is to investigate their fatigue performance. Fatigue tests involving FRP and SMA reinforcement have been conducted in few studies [42,43,44,45,46]. An experimental study [42] was conducted to examine the flexural behavior of corroded steel RC beams strengthened with CFRP sheets under repeated loading. The results revealed that the steel bars are the controlling factor of the fatigue capacity of RC beams. Therefore, repairing the beam or replacing steel with innovative materials like FRP was the way to increase the capacity of the beam with corroded steel reinforcement. After repairing and external CFRP-strengthening of RC corroded beam for flexure, the fatigue capacity was higher than that of the control beam which had no corrosion. Additionally, Aidoo et al. [43] found that the fatigue capacity increase was also controlled by the quality of the bond between the CFRP sheets and concrete. In another study [44], concrete beams were strengthened with CFRP sheets and laminates and subjected to fatigue load. Two of the specimens were strengthened with precured laminates attached to the concrete surface with epoxy adhesive or mechanical fasteners. Two other specimens were strengthened using CFRP sheets and spikes as anchorage. The results revealed that both epoxy and mechanical fasteners can be equally effective in bonding the CFRP laminates and the mechanical fasteners can be an alternative to the epoxy adhesive. However, the epoxy bonded CFRP sheets with anchor spikes provided the highest ultimate strength for the beam as compared to mechanical fasteners, as well as sustain large number of fatigue cycles.

Yun et al. [45] conducted an experiment on RC beams strengthened with NSM FRP. They revealed that NSM is the eminent method among others (external bonding, fiber anchored bonding, and hybrid bonding) to sustain good bond between FRP and concrete surface under fatigue load.

As oppose to FRP, the SMAs’ phases play a considerable role in strengthening RC elements to sustain the fatigue load [46]. If SMA is in austenite state (high temperature phase) the SMA bars undergo considerable deformation, while in martensite state (cooling phase), there is no residual deformation after the removal of the load. Consequently, SMA can provide outstanding fatigue resistance capabilities and large energy dissipation compared to steel.

The combination of noncorrosive property and high strength capacity makes the FRP an outstanding material as an internal and external reinforcement in RC structures. The durability of FRP is related to the fiber types (carbon, glass, basalt etc.) and epoxy resin matrix used [47]. Moreover, carbon fibers are more durable than glass fibers in harsh environment [47,48].

While the upfront cost of FRP materials might be higher than the steel, the maintenance and damage repair expenses would be reduced over the life of structures when using FRP as an internal and external reinforcement [49].

As oppose to FRP, limited use of SMA has always been associated with high cost of its composition of Nickel and Titanium (Ni-Ti). However, shaping the alloys by different composite materials like Cu-Zn-Al, and Cu-Al-Ni resulted in a decrease in cost ratio while providing comparable properties to traditional SMA composition. Depending on the shape and required material quantities, the cost ratios range from 1 to 10 for Cu-Zn-Al and from 2 to 20 for Cu-Al-Ni, while it ranges from 10 to 100 when Ni-Ti material is used. From this analysis, a significant reduction in cost was observed when the Ti was removed and Ni was replaced with another component, such as Zn [50].

## 4. Conclusions

The performance of concrete BCJs reinforced with innovative smart and corrosion free materials such as FRP, SMA, and hybrid and composite SMA-FRP are discussed in this paper. Based on the present literature review conducted in this study on RC BCJs, the following conclusions are drawn in the order of internal, external reinforcement of the BCJs and the effect of durability and cost.

- BCJs reinforced with FRP, SMA, and hybrid FRP-SMA longitudinal bars and stirrups performed better in improving overall load carrying capacity as compared to their counterpart one reinforced with traditional steel bars.
- At the same drift ratio, composite SMA-FRP BCJs bars showed higher ductility and energy dissipation than its counterpart reinforced with GFRP only.
- Under the same PGA values, GFRP reinforced BCJs exhibited higher drift ratio as compared to the counterpart composite SMA-FRP reinforced joints. However, joints reinforced with SMA-FRP bars sustained small amount of residual displacements in contrast to the ones with steel and GFRP.
- The performance of hybrid SMA-FRP reinforced BCJs under seismic loading was enhanced, specifically in the plastic hinge zone region. Furthermore, considerable improvements in ductility, residual drift, and energy dissipation were observed when compared to the joints reinforced with GFRP bars.
- Even though, in some cases, GFRP reinforced joints showed ductile behavior under reversed cyclic loading, the performance of traditional steel or hybrid SMA-steel reinforced joints were superior under earthquake loading particularly in terms of load carrying capacity and residual displacement.
- To assure the occurrence of plastic hinge away from the joint following the capacity design rule (strong column weak beam concept), sufficient anchorage is needed in the FRP-reinforced joint core region. While for hybrid SMA-FRP reinforced joints, placement of SMA bars in the joint core is essential.
- FRP and SMA materials can offer unique replacement for steel reinforcement bars due to their noncorrosive property along with the high strength. In the case of FRP-SMA or SMA reinforcement, small residual displacements can also be achieved. This will result in less repair and maintenance and lower life cycle cost.
- For externally reinforced BCJs, more layers of FRP sheets provided higher joint’s load-carrying capacity.
- Using steel elements such as plates or angles, as anchorages for the FRP sheets, prevented FRP sheets from debonding, as well as increased the joint capacities (e.g., ductility, stiffness and energy dissipation).
- In some cases, FRP external strengthening could change the mode of failure from joint shear failure to flexural failure of the beam.
- In general, it was noted that for the same amount of reinforcement ratio, CFRP sheets confined the joint core more effectively than GFRP sheets.
- The increase of column’s axial load helped in enhancing the load-carrying capacity and stiffness of the joints in the case of both internal and external FRP reinforcement.
- Strengthening with FRP and SMA helped in improving the fatigue capacity while providing protection against harsh environment and corrosion for RC beams.
- Although the high cost of SMA material limits its field applications for strengthening old or new structures with SMA elements; replacing Ni-Ti traditional SMA with other compositions such as Cu-Zn-Al, and Cu-Al-Ni of the same shape and quantity, makes it more economical while providing comparable results. Whereas, using FRP as external reinforcement reduces the maintenance cost in the long term.

## 5. Recommendations and Future Work

Significant research work conducted on FRP and SMA in the past few decades has paved the way to utilize such smart materials as reinforcement in concrete structures. The most important factor for potential use of FRP and SMA is their cost which could possibly limit the wider applications of such materials in the field of structural engineering. In the last decade, the price of SMA material has significantly decreased up to considerable amount due to its new composition. It is expected that the price will further reduce once it becomes widely and commonly used like FRPs. The application of SMA in RC structures will not only increase the cost due to material, but also costs associated with equipment and labor charges. However, FRP and SMA materials have superior quality to resist corrosion. In RC structures, the use of SMA in the plastic hinge zones could reduce cracks formation. Due to the re-centering capability, SMA could regain its original shape and size after experiencing larger inelastic deformations under high seismic loadings thereby assuring serviceability and reduction in maintenance of the structure. The effectiveness of FRP and SMA is exceptional in minimizing risks to human life associated with unpredicted natural disaster events. Further research could be carried out by using FRP and SMA in structures subjected to blast and fire. Moreover, coupling SMA with FRP and steel bars could also be effective in providing ductility.

Recently, NSM has become a popular method of externally strengthen RC elements, such as beams and columns, with FRP bars; however, the study of NSM technique on the BCJs can further be investigated.

Furthermore, the use of steel elements such as plates or angles as anchorages for the FRP sheets can be dispensed in future studies to prevent the joints from corrosion, since the FRP material is non-corrosive.

## Figures and Tables

**Table 1:** Summary of experimental work on BCJs internally reinforced with FRP.

BCJs	[7]		[8]		[9]		[10]		[11]		[12]	
	Beam	Column	Beam	Column	Beam	Column	Beam	Column	Beam	Column	Beam	Column
Test parameters	Longitudinal and lateral reinforcement types		Concrete strength and beam and column longitudinal reinforcement		Longitudinal reinforcement and stirrups types and ratios		Longitudinal reinforcement and stirrups types, and details of beam longitudinal reinforcement		Concrete strength, and joint shear stress level		Presence of lateral beams, and joint shear stress level	
Cross-section (mm^2^)	250 × 400	400 × 250	150 × 200	150 × 200	350 × 450	350 × 500	350 × 450	350 × 350	350 × 450	400 × 350	300 × 350	350 × 400
GFRP longitudinal reinforcement	8#16	12#16	4#12	4#12	5#19	8#19	5#16	8#16	8#16	12#16	10#16	12#16
GFRP transversal reinforcement (leg/mm)	3#10 @ 80 mm	3#10 @ 80 mm	2#8 @ 150 mm	2#8 @ 150 mm	3#13 @ 100 mm	3#13 @ 90 mm + 1 transversal #10	3#13 @ 100 mm	3#13 @ 90mm	3#12 @ 100 mm	3#12 @ 90 mm	3#12 @ 100 mm	3#12 @ 90 mm
Tensile strength for longitudinal & transversal bars (MPa)	600		580		590	#13 = 590,	751		1008		1100	
						#10 = 642						
Concrete compressive strength (MPa)	_		44.15		30		32.5		51.3		42.2	
Load type	Cyclic loading	Constant axial load of 600 kN	Monotonic loading	Constant axial load	Cyclic loading	Constant axial load of 800 kN	Seismic loading	Constant axial service load = 15% column capacity	Cyclic loading	Constant axial service load = 15% column capacity	Cyclic loading	Constant axial service load = 15% column capacity
Max. lateral load capacity (kN)	120		13.2		150		-		150		170	
Drift ratio (DR) at max. load lateral capacity (%)	6		5		4		4		6		6	
Peculiarity	Using GFRP grid instead of bars and stirrups		Steel bend couplers at the joint for GFRP bars				200 mm long beam stub		Couplers to connect bars together		Lateral beams as confinement	
Cumulative energy dissipation (kN·m)	60		_		58 at 5% DR		23 at 5% DR		28		50	
Observed failure mode	Specimens were not tested up to failure		Joint shear failure		Concrete crushing at 4% DR in beam + rupture of beam GFRP bars at 5% DR		Concrete compression failure		Plastic hinge in the beam section followed by slippage of longitudinal reinforcement		Buckling of beam bars	

**Table 2:** Summary of experimental work on BCJs externally reinforced with FRP using steel anchorages.

BCJs	[25]		[26]		[28]		[37]		[40]	
	Beam	Column	Beam	Column	Beam	Column	Beam	Column	Beam	Column
Type of study	Experimental study		Experimental study		Experimental study		Experimental study		Experimental study	
Number of specimens	4 NS BCJs: 2 damaged + strengthened, 2 strengthened		2 NS BCJs: 1 damaged + strengthened, 1 strengthened		4BCJs non-seismic 1/2 scale: 2 control, damaged + strengthened, 2 strengthened with CFRP		6 NS BCJs: (a) 3 anchorage deficient: 1 control + 2 strengthened, (b) 3 shear anchorage deficient: 1 control + strengthened		8 full-scale BCJ: 2 control + 6 strengthened	
Test parameters	number of GFRP layers, joint height to be repaired, shape of sheets applied		number of GFRP layers, shape of sheets applied		CFRP placing areas		inadequate anchorage length of beam bottom bars, absence of steel ties in the joint zone, type of FRP, anchorage systems(steel plates, angles, rods), steel reinforcement ratio		steel reinforcement ratio, joint strengthening configuration, damaged and undamaged	
Cross-section (mm^2^)	250 × 400	400 × 250	250 × 400	400 × 250	160 × 350	300 × 160	250 × 400	400 × 250	300 × 400	300 × 300
Internal main reinforcement	4 Ø 20 top + 4 Ø 20 bottom	6 Ø 20 + 2 Ø 15	4 Ø 20 top + 4 Ø 20 bottom	6 Ø 20 + 2 Ø 15	4 Ø 12 top + 4 Ø 12 bottom	10 Ø 10	(a) 2 Ø 20 top + 2 Ø 20 bottom, (b) 4 Ø 20 top + 4 Ø 20 bottom	(a) (b) 6 Ø 20 + 2 Ø 15	1–3 Ø 20 top + 3 Ø 20 bottom, 2–4 Ø 20 top + 4 Ø 20 bottom	1–4 Ø 14, 2–8 Ø 14
Internal shear reinforcement (leg/mm)	2 Ø 10 @ 150 mm	3 Ø 10 @ 200 mm	2 Ø 10 @ 150 mm	3 Ø 10 @ 200 mm	2 Ø 6 @ 225 mm	2 Ø 6 @ 140 mm	2 Ø 10 @ 150 mm	3 Ø 10 @ 200 mm	2 Ø 8 @ 100 mm	2 Ø 8 @ 100 mm
Reinf. yield strength (MPa)	#10: 450, #15: 408, #20: 425		#10: 450, #15: 408, #20: 425		420		#10: 450, #15: 408, #20: 425		540	
FRP type	GFRP		GFRP		CFRP		(a) CFRP, (b) GFRP		CFRP	
FRP shape and configuration	bi-directional U-shaped + unidirectional X-shaped GFRP sheets		uni and bi-directional U-shaped GFRP sheets		unidirectional CFRP sheets		uni and bi-directional GFRP sheets		horizontal sheets, X-shaped, U-shaped	
FRP thickness (mm)	-		bi-directional = 0.864, unidirectional =0.353		1		-		0.22	
Number of FRP layers	1, 2, 3		2, 4, 8		1		2, 4, 8		1, 2	
FRP tensile strength	-		bi-directional = 279 MPa, unidirectional = 1700 MPa		-		CFRP = 709.6 N/mm per sheet‘s width, uni-directional: 465.0 N/mm per sheet’s width		3000 MPa	
Concrete compressive strength (MPa)	25		30.6, 43.5, 39.5 avg. 37.8		group 1: 30, group 2: 25		-		16	
Load type	constant axial column load (300,600 kN) + reversing quasi-static cyclic load		constant 600 kN axial column load + reversing quasi-static cyclic load		cyclic loading by a 500 kN actuator + constant axial load on the column = 20% of column’s capacity		constant 600 kN axial column load + cyclic load		cyclic loading	
Peculiarity	column confined by GFRP sheets and anchored using steel plates and threaded steel rods		column confined by GFRP sheets and anchored using steel plates and threaded steel rods + beam GFRP sheets anchored with U-shaped steel plates		restrained with slab, 2 schemes for strengthening, 2nd scheme uses bolts and steel plates to provide mechanical anchorages		(a) CFRP sheets on columns anchored using steel plates and threaded steel rods + beam CFRP sheets anchored with U-shaped steel plates, (b)one of BCJ steel rods threaded from column towards beam and covered with epoxy mortar, tie rods welded to beam bottom bars		use of steel plates on the beam, threaded rods, L-shaped and U-shaped steel plates	
Max. lateral load capacity (kN)	127		damaged-strengthened: 110, undamaged-strengthened: 114.4		85.4 in undamaged-strengthened specimens		(a) 86, (b) 152		push: avg. 76.2, pull: avg. 73.1	
DR of max. capacity (%)	-		Damaged-strengthened 1.2, undamaged-strengthened 1.3		-		(a) 1.98, (b) 2.43		push: avg. 1.74, pull: avg. 1.73	
Ductility increase as compared to control (%)	60		higher than control		Increase: damaged-strengthened avg. 36, undamaged-strengthened 66.5		higher than control		72	
Cumulative energy dissipation increase as compared to control (%)	72		300 for damaged-strengthened and 600 for undamaged-strengthened		-		-		slightly higher than control	
Observed failure mode	shear failure of the joint and flexural hinging of the beam		shear failure of the joint		control: shear failure, strengthened: scheme1: debonding, scheme2: crushing of beam’s concrete, strengthened: debonding and crushing beam’s concrete		(a) debonding, beam hinging. (b) shear of rehab section, fracture of rehab rods		Small FRP delamination followed by joint shear failure	
Other results	-		-		shear strength was improved		-		-	

**Table 3:** Summary of experimental work on full-scaled BCJs externally reinforced with one FRP type.

**BCJs**	**[1]**		**[6]**		**[29]**		**[38]**	
	**Beam**	**Column**	**Beam**	**Column**	**Beam**	**Column**	**Beam**	**Column**
**Type of study**	**Experimental study**		**Experimental study**		**Experimental study**		**Experimental study**	
Number of specimens	6BCJs full scale: 3 controls, 3 wrapped with CFRP		1BCJ repaired + 3 full-scale BCJs strengthened with CFRP		2BCJs		6 full-scale corner BCJ	
Test parameters	column reinf. configuration, num. of layers + CFRP wrapping configuration		types of beam anchorage detailing		number of CFRP sheets		presence of CFRP strengthening under the slab, fibers orientation, CFRP thickness, column confinement with CFRP sheets, concrete compressive strength	
Cross-section (mm^2^)	300 × 500	300 × 300	260 × 600	260 × 260	350 × 400	350 × 350	(a) 300 × 500, (b) stub: 300 × 500	300 × 300
Internal main reinforcement	3 Ø 20 top + 2 Ø 20 bottom	6 Ø 20 continuous or with lap splicing	4 Ø 16 top + 4 Ø 16 bottom	top part: 4 Ø 16, bot part: 8 Ø 16	4 Ø 18 top + 4 Ø 18 bottom	12 Ø 18	(a) 5 Ø 16 top + 3 Ø 16 bottom, (b) 3 Ø 16 top + 2 Ø 16 bottom	4Ø16
Internal shear reinf. (leg/mm)	2 Ø 10 @ 150 mm + 2 Ø 10 @ 75 mm in tip	2 Ø 10 @ 75 mm	2 Ø 8 @ 150 mm	2 Ø 8 @ 150 mm	2 Ø 10 @ 100 mm	2 Ø 10 @ 100mm	Ø8 @ 200 mm	Ø8 @ 200 mm
Reinf. yield strength (MPa)	420		Ø16: 551, Ø8: 612		Ø18: 417, Ø10: 282		470	
FRP type	CFRP		CFRP		CFRP		CFRP	
FRP shape and configuration	unidirectional CFRP U-shaped and straight sheets and strips		Sheets		unidirectional CFRP sheets		unidirectional and quadriaxial sheets	
FRP thickness (mm)	0.176		0.185		-		0.053	
Number of FRP layers	1, 2, 3		1, 2, 3		1, 2, 3		1, 2	
FRP tensile strength (MPa)	3800		4140		4100		Uniaxial = 2540, quadriaxial = 3450	
Concrete compressive strength (MPa)	26	24	<15, 15 to 20		24.25 and 19.60		avg. 25.5	
Load type	cyclic loading on column tip + 700 kN const. axial load		reversed cyclic loading + 150 kN column axial load		cyclic loading		Cyclic loading	
Peculiarity	CFRP belts to stop the slippage of longitudinal beam bot. reinf. + to prevent debonding of the U-shaped beam’s wraps		replacing the damaged concrete with high-strength-concrete in the joint core		steel angles on top and bottom face of the beam to carry the tensile forces to column		presence of slab	
Max. lateral load capacity	avg. increase: pull 61%, push: 118%		rehab.: increase 44%, strengthened: increase avg. 108%		avg. increase: pull 17%, push 32%		Increased: 96%	
DR of max. capacity (%)	control avg. pull = 1.11, push = 1.1. wrapped avg. pull = 1.72, push = 2.35		rehab.: 2		avg.: pull increase 44, push decrease 34		2.3	
Ductility			-		increased by 97% and 50%		did not significantly increase	
Cumulative energy dissipation	control avg.7.6 kN·m, disp. 35 mm. wrapped avg. 33 kN·m, disp. 69 mm		strengthened higher than control		1st BCJ decrease 15%, 2nd BCJ increase 60%		increased by 31.5%	
Observed failure mode	control: joint shear + debonding of beam bottom bar. wrapped: debonding + the rupture of CFRP sheets at the beam faces out of joint		control and rehab.: joint shear failure, strengthened: rupture of CFRP sheets		CFRP debonding and rupture		CFRP debonding with joint shear failure, or with column flexural hinging, flexural hinging with CFRP rupture	
Other results	delay in stiffness degradation of CFRP BCJ compared to control BCJ		-		using steel angles with round corners helped decrease the stress concentration on corners		-	
**BCJs**	**[30]**		**[32]**		**[39]**			
	**Beam**	**Column**	**Beam**	**Column**	**Beam**	**Column**		
**Type of study**	**Experimental study**		**Experimental study**		**Experimental study**			
Number of specimens	3 NS BCJs strengthened, 1 control NS		1 full-scale BCJ tested and repaired with CFRP		1 RC structure with multiple joints			
Test parameters	number of CFRP layers		repaired and not repaired		column dimensions, GFRP configurations PGA level (a—0.2, b—0.3 g)			
Cross-section (mm^2^)	200 × 200	167 × 167	250 × 500	500 × 250	250 × 500	(a) 8 col.s: 250 × 250, (b) 1 col.: 250 × 750		
Internal main reinforcement	6 Ø 16	6 Ø 10	5 Ø 18 top + 5 Ø 18 bottom	8 Ø 18	Ø12 and Ø20	(a) 4 Ø 12, (b) 10 Ø 12		
Internal shear reinf. (leg)	2 Ø 10 @ 100 mm	2 Ø 4 @ 134 mm	2 Ø 10 @ 100 mm	2 Ø 10 @ 150 mm	Ø8 @ 200 mm	Ø8 @ 250 mm		
Reinf. yield strength (MPa)	Ø16: 550, Ø12: 551, Ø10: 322		292.5		smooth reinf.: 320			
FRP type	CFRP		CFRP		GFRP			
FRP shape and configuration	different ratios of CFRP sheets		flat and X-shaped sheets		unidirectional and quadriaxial laminates			
FRP thickness (mm)	0.11		-		uni-axial = 0.48, quadriaxial = 0.1096			
Number of FRP layers	1, 2, 3, 6, 12		-		-			
FRP tensile strength (MPa)	-		-		uni-axial =1314, quadriaxial= 986			
Concrete compressive strength (MPa)	40		8.05		avg. 25.5			
Load type	cyclic loading by a 600 kN hydraulic actuator		Quasi static cyclic loading		pseudo-dynamic			
Peculiarity	modify the column square shape to round shape before wrapping the CFRP sheets		use low concrete strength, and plain round reinf. bars, weld the beam hooks with column main reinforcement		-			
Max. lateral load capacity	increase by avg. 113%		decrease: pull 22%, push 25%		*x* direction: 11%, *y* direction: 7.7%			
DR of max. capacity(mm)	-		-		top displacement: *x* direction: a—108.8 mm, b—205.3, *y* direction: a—112.5 mm, b—126.6 mm			
ductility	-		decreased		-			
Cumulative energy dissipation as compared to control (%)	avg. increase 242		decrease 4		*x* direction: 182, *y* direction: increase 228			
Observed failure mode	Shear failure because the CFRP rupture, beam flexure failure far from column face		joint shear failure due to debonding of x-shaped sheets on the joint surface		N/A			
Other results			stiffness lower than the control specimen		column confinement increased the plastic hinge capacity			

**Table 4:** Summary of experimental work on scaled-down BCJs externally reinforced with FRP.

BCJs	[31]		[33]		[36]	
	Beam	Column	Beam	Column	Beam	Column
Type of study	Experimental and analytical study		Experimental study		Experimental study	
Number of specimens	4 NS BCJs damaged-strengthened, 1 control NS, and 1 (S) seismic		8BCJs 1/3 scale: 1 (NS) non-seismic, 1 (SD)seismic, 6 damaged+ strengthened		18BCJ 2/3 scale	
Test parameters	number of CFRP layers, loading drift ratio		configuration of CFRP sheets, the presence of strips		area fraction of FRP; distribution of FRP between the beam and the column; column axial load; internal joint steel reinforcement; initial damage; FRP type and configuration; effect of transverse beams	
Cross-section (mm^2^)	300 × 300	300 × 400	134 × 200	200 × 300	200 × 300	200 × 200
Internal main reinforcement	4 Ø 18 top + 4 Ø 18 bottom	8 Ø 18	4 Ø 10 top + 2 Ø 10bottom	4 Ø 12 top + 4 Ø 12 bottom	3 Ø 14 top + 3 Ø 14 bottom	4 Ø 14
Internal shear reinforcement (leg)	S: 2 Ø 10 @ 70 mm NS: 2 Ø 10 @ 150 mm		NS: 2 Ø 4 @ 87 mm, S: 2 Ø 4 @ 44 mm	2 Ø 10 @ 75 mm	2 Ø 8 @ 150 mm	2 Ø 8 @ 100 mm
Reinforcement yield strength (MPa)	Ø18: 533.3, Ø10: 509.9		459		longitudinal: 585, shear: 220	
FRP type	CFRP		CFRP		CFRP, GFRP	
FRP shape and configuration	L-shaped, U-shaped and flat-shaped CFRP laminates		T-shaped, L-shaped, X-shaped, strips		sheets, strips	
FRP thickness (mm)	0.167		0.33		CFRP = 0.13, GFRP = 0.17	
Number of FRP layers	1, 2		1, 2		2, 3, 4, 5, 6	
FRP tensile strength (MPa)	4950		4965.8		CFRP strips = 2400, CFRP sheets = 3450, GFRP sheets = 2170	
Concrete compressive strength (MPa)	38.6		36.5		avg. 25	
Load type	reversed cyclic loading		cyclic loading by a 500 kN actuator applied on tip of column		simulated seismic load	
Peculiarity	-		-		use of lateral beam, use of anchorages with or without the lateral beam	
Max. lateral load capacity (kN)	improved or equal to the control one		11.27 kN with an avg. increment of 18.22% compared to NS non-retrofitted specimen		without transverse beam: avg. 43.5 kN, with transverse beam = avg. 42.1 kN	
DR of max. capacity (%)	-		-		Slightly increased	
Ductility	-		Avg. of 9.24 kN·m		-	
Cumulative energy dissipation	-		-		increase 70–80%	
Observed failure mode	N/A		shear failure for NS, beam flexure failure for CFRP-strengthened		FRP debonding	
Other results	the stiffness decreased even after repair and strengthening compared to NS specimen. the damage could be repaired until 1.5% DR		increase in number of sheets increased the strength. The X-shaped was better in improving the strength		2.5 times higher column axial load than the initial load is the most effective load, anchorages increased strength by 30% and energy dissipation by 40%	

**Table 5:** Summary of experimental work on BCJs externally reinforced with more than one FRP types (CFRP, GFRP).

BCJs	[5]		[27]	
	Beam	Column	Beam	Column
Type of study	Experimental study		Experimental study	
Number of specimens	4BCJs non-seismic control and then repaired + strengthened		12BCJs: 6 NS + 6 SD	
Test parameters	beam and column cross sections, number of FRP sheets, column axial load		FRP types, and layers	
Cross-section (mm^2^)	(1): 230 × 300, (2): 230 × 600	(1): 280 × 820, (2): 300 × 1600	100 × 100	100 × 100
Internal main reinforcement	(1): 2 Ø 13 top + 2 Ø 13 bottom, (2): 3 Ø 25 top + 3 Ø 20 bottom	(1): 4 Ø 25 + 4 Ø 22, (2): 24 Ø 28	4 Ø 6	4 Ø 6
Internal shear reinforcement (leg/mm)	-	-	2 Ø 3 @ 100 mm	2 Ø 3 @ 100 mm
Reinf. yield strength (MPa)	510		555.13	
FRP type	GFRP + CFRP		GFRP + CFRP	
FRP shape and configuration	uni-directional sheets of GFRP and CFRP, L-shaped + U-shaped		GFRP + CFRP sheets, CFRP plates (CP)	
FRP thickness (mm)	GFRP: 1.3, CFRP: 1.0		GFRP = 0.36, CFRP = 0.11, CP = 1.2	
Number of FRP layers	1, 2		1, 2	
FRP tensile strength (MPa)	GFRP = 575, CFRP = 986		GFRP = 2250, CFRP = 3500	
Concrete compressive strength (MPa)	19.5		30	
Load type	quasi-static loading		cyclic loading + 100 kN constant axial load on column	
Peculiarity	-		use carbon plates in one of the strengthening schemes	
Max. lateral load capacity as compared to control (%)	(1): avg. increase 52, (2): same capacity		increased in strengthened joints	
DR of max. capacity	(1): 3%, (2): 4%		-	
Ductility	-		SD joints were more ductile than NS joints	
Cumulative energy dissipation (%)	(1): recovered 74% of its original energy, (2): recovered 76.5% of its original energy		SD: avg. 111, NS: avg. 122	
Observed failure mode	control: (1): plastic hinge in beam + (2): plastic hinge penetrated joint. repaired-strengthened: debonding and delamination.		-	
Other results	series (1) maintained higher stiffness, series (2) restore same stiffness		-	

## Abbreviations

The following abbreviations are used in this manuscript:

NSM	near surface mounting
FRP	fiber reinforced polymers
GFRP	glass fiber reinforced polymers
CFRP	carbon fiber reinforced polymers
RC	reinforced concrete
NS	non-seismically designed
SD	seismically designed
PGA	peak ground acceleration
MRFs	moment resisting frames
Ids	inter-storey drifts
BCJs	Beam-Column Joints
NS	non-seismically
SD	Seismically designed
RNS	Retrofitted non-seismically designed
